# A Micromachined Metal Oxide Composite Dual Gas Sensor System for Principal Component Analysis-Based Multi-Monitoring of Noxious Gas Mixtures

**DOI:** 10.3390/mi11010024

**Published:** 2019-12-24

**Authors:** In-Hwan Yang, Joon-Hyung Jin, Nam Ki Min

**Affiliations:** 1 Department of Chemical Engineering, Kyonggi University, 154-42 Gwanggyosan-ro, Yeongtong-gu, Suwon-si, Gyeonggi-do 16227, Korea; ihyang@kgu.ac.kr; 2 Department of Electro-Mechanical Systems Engineering, Korea University, 2511 Sejong-ro, Sejong City 30019, Korea

**Keywords:** dual-sensor system, microelectromechanical systems, metal oxide nanocomposite, noxious gas mixture

## Abstract

Microelectronic gas-sensor devices were developed for the detection of carbon monoxide (CO), nitrogen dioxides (NO_2_), ammonia (NH_3_) and formaldehyde (HCHO), and their gas-sensing characteristics in six different binary gas systems were examined using pattern-recognition methods. Four nanosized gas-sensing materials for these target gases, i.e., Pd-SnO_2_ for CO, In_2_O_3_ for NO_X_, Ru-WO_3_ for NH_3_, and SnO_2_-ZnO for HCHO, were synthesized using a sol-gel method, and sensor devices were fabricated using a microsensor platform. Principal component analysis of the experimental data from the microelectromechanical systems gas-sensor arrays under exposure to single gases and their mixtures indicated that identification of each individual gas in the mixture was successful. Additionally, the gas-sensing behavior toward the mixed gas indicated that the traditional adsorption and desorption mechanism of the n-type metal oxide semiconductor (MOS) governs the sensing mechanism of the mixed gas systems.

## 1. Introduction

With the increasing importance of indoor and outdoor air quality for human health and the natural environment, various air-monitoring applications equipped with highly sensitive gas sensors have been used for detecting hazardous gases emitted from various sources, such as automobiles, industrial plants, waste-management facilities, and food and household products. In the past decade, metal oxide semiconductor (MOS)-based gas sensors have attracted considerable attention owing to their high compatibility with various consumer applications that demand low energy consumption, simplicity of use, and multi-gas detection ability [1]. MOS gas sensors, which can detect either the oxidation or reduction of gas coming into contact with a thin metal oxide surface by measuring changes in the surface conductivity, have been successfully manufactured using thin- and thick-film technologies for the microfabrication of a gas-sensitive metal oxide film on an electrode substrate [2,3,4,5]. However, their low selectivity, response-curve drifts, temperature-dependent properties prevent them from achieving high selectivity and sensitivity to the target gas species in a gaseous atmosphere with long-term stability and reliability [6,7]. Besides these intrinsic sensing properties of metal oxide materials, environmental conditions such as the presence of water and interference gas species in an atmosphere also affect the selectivity and sensitivity of MOS sensors [8].

To overcome these issues related to selectivity and sensitivity, nanomaterials are increasingly used as sensing materials for MOS sensors owing to their size-dependent properties. In principle, the sensing response is highly dependent on the amount of available surface active sites, because the gas contacting the sensing material is detected via a process involving gas adsorption, charge transfer, and desorption on the surface of the sensing materials. Nanomaterials, which have a large surface-area-to-volume ratio, can significantly increase the number of adsorbed gas molecules participating in the oxidation or reduction reactions on the surface of the sensing materials, as well as the interfacial transport of electrons and holes. Nanosized metal oxide materials doped with noble metals or synthesized with mixed metal oxides improve the sensitivity and selectivity to a target gas through the catalytic reactivity and morphology of deposited films [9,10].

In addition to the use of the advantages of nanomaterials as sensing materials for MOS sensors, multivariate calibration techniques that employ transient sensor responses to changes such as in sensor temperature can also improve the sensitivity and selectivity of MOS sensors regardless of sensing material [11,12]. A temperature variation applied to a single MOS sensor generates a unique response pattern for a reactive gas species owing to the different reaction rates of the gaseous species at temperatures. The obtained signal pattern is therefore used to identify a target gas species from an ambient atmosphere. This multivariate analysis of sensing data had led to apply an array design to further improve the sensitivity and selectivity of MOS sensors. A sensor array comprising individual sensors with different characteristics is used in conjunction with pattern-recognition techniques [7,13]. For a multi-sensor array to have spatial gas-sensitive properties, a single metal oxide film segmented by electrodes is coated with a varied-thickness membrane, and/or a temperature gradient is induced along the array. The different thicknesses of the membrane and/or the controlled temperature of each segmented element in the MOS sensor array can allow individual MOS sensors to obtain partial gas sensor responses that are independent from the other elements of the sensor array. A group of response signals continuously delivered from the MOS sensor array is analyzed via pattern-recognition techniques for characterizing and quantifying gases to which the array is exposed. However, such multi-sensor arrays suffer from contaminant doping in the sensing area during the manufacturing process, which unpredictably alters the resistance of the sensing elements in the sensor array [14,15].

In this study, we developed a dual-sensor system in which two different individual sensors were assembled into an array for monitoring multi-gas species. Individual sensors used for the dual-sensor system were equipped with different nanocomposites as sensing materials to enhance its selectivity and sensitivity. Independent output signals obtained from the dual-sensor system were analyzed by using a multivariate analysis method to identify different gas species in an atmosphere. Multi-gas sensing properties of the developed dual-sensor system were assessed by using four different noxious gas species: carbon monoxide (CO), nitrogen oxides (NO_2_), ammonia (NH_3_), and formaldehyde (HCHO). Each gas-sensor module of the dual-sensor system was designed to allow the operating temperature of the sensing nanocomposite to be controlled by a micro-electromechanical systems (MEMS)-based platform for the sensing elements, where a microheater and sensing electrode were constructed together on the coplanar surface of a silicon substrate via microfabrication processes [16,17]. The changes of the conductivities of the dual MOS gas sensor exposed to atmospheric gases including diluted toxic gas mixtures were measured and subjected to principal component analysis (PCA) to identify individual toxic gases. The PCA plots for binary toxic gas mixtures were obtained to discriminate each toxic gas from the atmospheric gases. The PCA results indicate that using metal oxide nanocomposites at the optimized temperatures enhanced the sensitivity and selectivity of the dual-sensor system for multi-detecting and multi-monitoring the low concentrations of each toxic gas species in the atmospheric gas.

## 2. Materials and Methods

### 2.1. Fabrication of Metal Oxide Semiconductor (MOS) Sensor Modules

**Figure 1:**
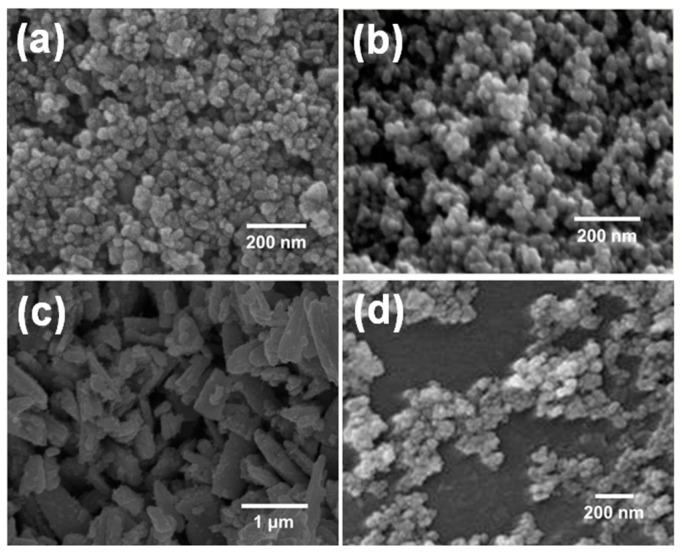
Microstructures of the four gas-sensing materials: (**a**) Pd-doped SnO_2_; (**b**) In_2_O_3_; (**c**) Ru-doped WO_3_; (**d**) Pd-doped SnO_2_-ZnO.

Gas-sensing elements for the detection of the target gases, i.e., SnO_2_ for CO, In_2_O_3_ for NO_X_, WO_3_ for NH_3_, and SnO_2_-ZnO for HCHO, were synthesized via the conventional sol-gel process [16,18,19,20]. Each sol carefully mixed with the appropriate sensing material was dropped and dispensed on the electrode substrate of the sensor platform, and then the deposited sensing material was heated at the appropriate sintering temperature [21]. Approximately 1 wt % Pd nanopowder was added to the SnO_2_ and the SnO_2_-ZnO composite to increase the electrical conductivity without sacrificing the selectivity to the targets. Approximately 1 wt % Ru nanopowder was added to WO_3_ for the same reason. Figure 1 shows field-emission scanning electron microscopy images of the nanomaterials used for the MOS gas-sensor module. The average particle sizes for the SN (SnO_2_ with 1 wt % Pd), IN (In_2_O_3_), WO (WO_3_ with 1 wt % Ru), and SZ (SnO_2_-ZnO with 1 wt % Pd) sensors were 40, 70, 1000, and 20 nm, respectively. The operating temperatures of the sensing materials were 267, 267, 334, and 367 °C, respectively.

**Figure 2:**
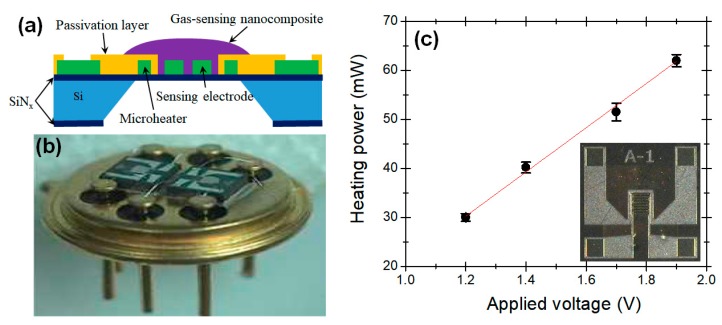
Physical characterization of the fabricated dual-sensor module: (**a**) cross-sectional view of the arrayed gas-sensor package, (**b**) photograph of a dual-sensor module, (**c**) output power vs. applied voltage curve of the microheater.

Figure 2a,b shows a cross-sectional schematic of the micro-platform used for the MOS sensor module and a photograph of the two-arrayed TO-39-packaged sensor system, respectively. Using the design concept of coplanar platforms for MEMS-based gas sensors reported in a previous work [17], a 1.8 × 1.8-mm^2^ silicon substrate equipped with a 0.6 × 0.6-mm^2^ microheater was fabricated as a micro-platform for the sensing nanocomposites. A Pt thin film was patterned on the silicon nitride layer as a microheater and the sensing electrode, and then silicon nitride was deposited as a passivation layer for insulating the constructed microheater. In fabricating the microheater at the center of the micro-platform, a serpentine pattern was employed to minimize the power consumption and provide a stable heating temperature to the deposited sensing materials (inset of Figure 2c). The fabricated MEMS-based gas-sensor module exhibited low power dissipation, and the heating power consumption was linearly proportional to the operation temperature in the range of 200–350 °C (Figure 2c). The optimized operating temperatures were 225 °C (at a power consumption of 35.26 mW) for the SN and IN sensors and 360 °C (at 64.37 mW) for the WO and SZ sensors [16,19,20].

### 2.2. Measurements

**Figure 3:**
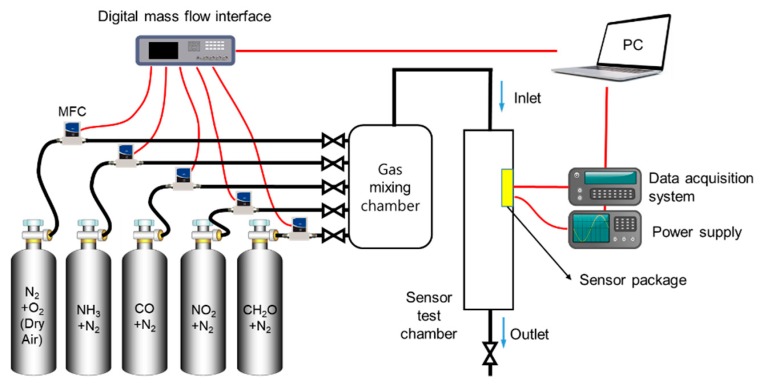
Schematic diagram of computer-controlled continuous gas flow system for sensor characterization.

A schematic diagram of a MOS sensor analysis system used in this study is shown in Figure 3. The analysis system mainly consists of a test chamber where the fabricated gas-sensor was placed, a control and data acquisition system, and a gas feeding system. Before measurements, a gaseous atmosphere in the test chamber with 2 L volume was maintained by flowing dry compressed air (N_2_ + O_2_) at a constant rate of 500 mL∙min^−1^, and then the micro-heater of the fabricated gas-sensor was heated at the optimized operating temperatures until a stable baseline was achieved. After that, the sensors in the chamber were exposed to an ambient gas flow composed of binary mixture gases and dry compressed air. The exposure time to a binary mixture gas and dry air was set up as 10 min, after which only dry air continuously flew to remove a binary mixture gas in the test chamber.

The gaseous atmosphere in the test chamber at room temperature was maintained by a computer-supervised gas flow system. During a typical sensing test, the concentration of target gases flowing in the test chamber was obtained by mixing a standard gas in nitrogen with certain amounts of dry compressed air using mass flow controllers (Line Tech, Daejeon, Korea). The total gas flow rate was set as 500 mL∙min^−1^. The concentration ranges of CO, NO_2_, NH_3_, and HCHO were 0–60 ppm, 0–0.6 ppm, 0–10 ppm, and 0–5 ppm, respectively. Note that concentration limits of NO_2_ and CO are 0.1 ppm and 35 ppm on the basis of 1-h exposure time considering the US environmental protection agency (EPA) standards, WHO Guidelines, and EU Air Quality Directive. Occupational Safety and Health Administration (OSHA) standards restrict the NH_3_ level in a working place to less than a short term exposure limit of 35 ppm during any 15-min period in the day. According to US EPA data, HCHO concentration of outdoor air in urban areas is between 10 and 20 ppb, and that of indoor is 0.1–3.68 ppm. Combining the individual noxious gases yielded six two-component gas mixtures. A laboratory-made transducer and network data acquisition unit (Fluke, Everett, WA, USA) was employed to record the real-time conductance changes of the gas sensor. The collected output signals from the mixed gas systems were linearly normalized and used as the input values of a data-processing algorithm for conducting PCA.

The gas sensitivity (*S*) is defined as *S* = log (*R*_g_/*R*_a_), where *R*_a_ represents the sensor resistance observed in the aerobic condition, and *R*_g_ represents the sensor resistance in the noxious gas environment. Therefore, a response of a dual-sensor system to a mixed gas system can be quantitatively compared with the response of another dual-sensor system to the same mixed gas system. Because the MOSs used in this study belonged to the n-type semiconductor group [22], the gas sensitivity would be negative (*S* < 0) if electron-rich gases such as CO, NH_3_, or HCHO were present in the surrounding area. Similarly, a positive sensitivity (*S* > 0) would be observed in the presence of an electron-deficient gas (NO_2_).

## 3. Results

### 3.1. Relative Sensitivity of Single Sensor Module

**Figure 4:**
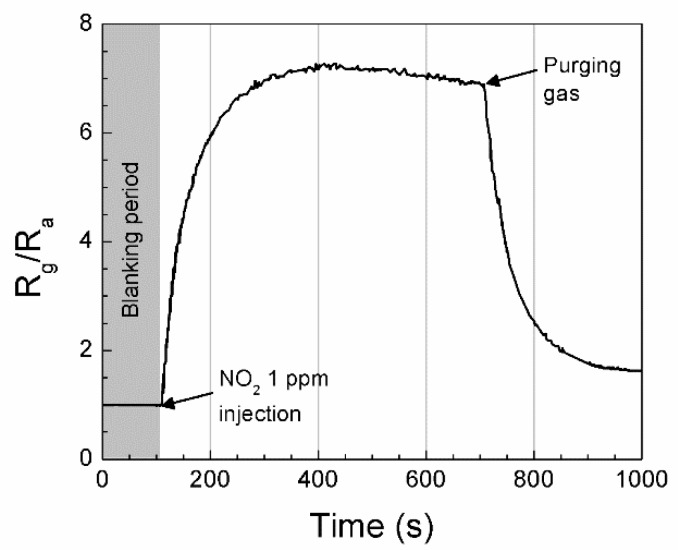
Response curve of the IN (In_2_O_3_) sensor for real-time motoring of NO_2_. The gas sensing process is composed of three steps, i.e., blanking period for 100 s, exposure to target gas for 600 s, and finally regeneration by dry air purging.

**Figure 5:**
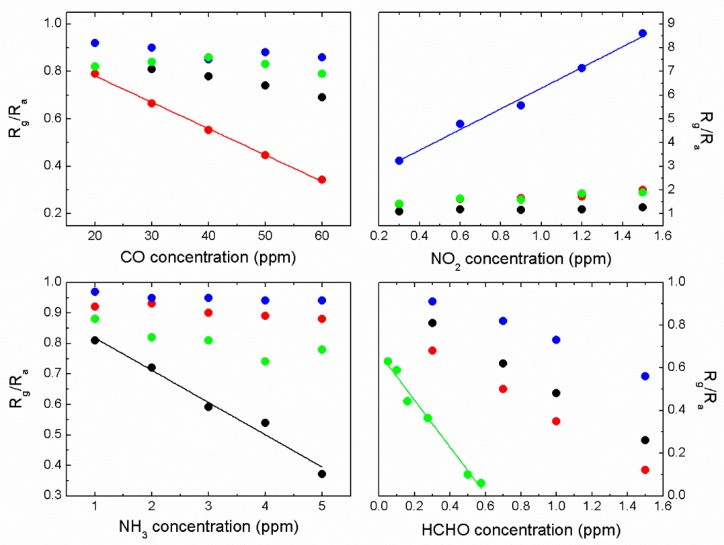
Calibration of the SN (SnO_2_ with 1 wt % Pd), IN, WO (WO_3_ with 1 wt % Ru), and SZ (SnO_2_-ZnO with 1 wt % Pd) sensors for monitoring CO, NO_2_, NH_3_, and HCHO. Note that black, red, blue, and green solid circles represent WO, SN, IN, and SZ, respectively.

The response characteristics of four individual sensor modules under an artificial noxious gas-contaminated aerobic condition were examined. Figure 4 shows a conventional response curve of a MOS sensor. The sensitivity *S* of the IN sensor for monitoring NO_2_, at first the *S* was 1 for blanking period, increased by the injection of 1 ppm NO_2_ into the test chamber and eventually saturated at around 7 in 200 s. Figure 5 shows the response curves of each single sensor module for monitoring all the target gases. The SN sensor exhibited a strong response to CO. The IN sensor was sensitive to NO_2_ and HCHO. Basically, the WO sensor exhibited responses to all the target gases and showed more sensitive to NH_3_ than the others. The SZ sensor was highly sensitive to HCHO. Even though we assumed that chemiresistive sensors shown in this work are working based on a conductivity change of n-type sensing material before and after adsorption of the target gases, there are many other factors affecting the conductivity change such as microstructure of the sensing materials including grain size and connectivity between the grains. The variation could become more serious if binary sensing materials were used.

Table 1 presents the preferential sensing responses of the individual sensor modules to two-component gas mixtures in atmospheric conditions. The results indicate that the electron-withdrawing NO_2_ gas elicited a strong response from all the sensors. In contrast, the electron-donating NH_3_ gas elicited a weak response. The IN sensor responded only to the NO_2_ gas among all the two-component mixture gases, and the SZ sensor exhibited the strongest response to HCHO, even in the NO_2_-HCHO mixture. This indicates that the surface interaction between sensing materials and the target gases, and the traditional oxygen-involved adsorption and desorption mechanism are collectively affecting the working mechanism of the sensor system, leading to an adsorption priority of the target gases [23].

### 3.2. Quantitative Approach to Investigate Response Characteristics of Dual-Sensor Modules

**Figure 6:**
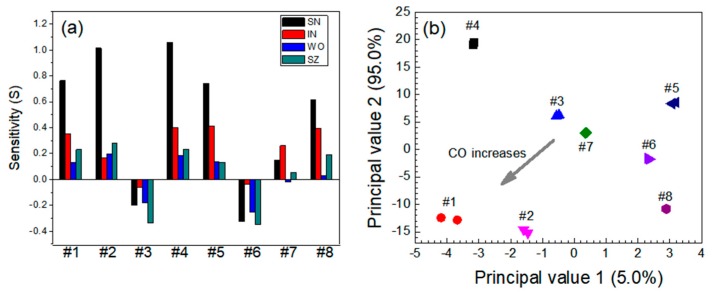
(**a**) Responses of the sensors and (**b**) principal component analysis (PCA)-based dual-gas sensor monitoring for the CO-NO_2_ system. Note that #1–#8 represent 30 ppm CO + 0 ppm NO_2_, 60 ppm CO + 0 ppm NO_2_, 0 ppm CO + 0.3 ppm NO_2_, 30 ppm CO + 0.3 ppm NO_2_, 60 ppm CO + 0.3 ppm NO_2_, 0 ppm CO + 0.6 ppm NO_2_, 30 ppm CO + 0.6 ppm NO_2_, and 60 ppm CO + 0.6 ppm NO_2_, respectively.

The sensing response obtained from each individual sensor module in a dual-sensor system should be as diverse as possible to ensure that the response of each sensor module to an exposed gas is non-correlated with the other sensor module in the system. These independent gas responses obtained from a dual-sensor system allow low concentrations of multi-species toxic gases in an atmospheric gas to be discriminated and identified. As an example, Figure 6a shows the response characteristics of all the single sensor modules to the CO-NO_2_ gas system. The gas sensitivity of all the sensor modules used in the experiments was *S* < 0 in the absence of CO in the mixture, whereas it was *S* > 0 in the presence of CO. For the CO-NO_2_ system, the gas response to CO, i.e., an oxidizing gas, was higher than that to the electron-donating NO_2_ gas for all the sensors. The SN sensor exhibited strong responses to both gases and their mixtures, whereas the IN sensor selectively responded to NO_2_. The WO and SZ sensors exhibited similar behaviors to the SN sensor, but their sensitivities to NO_2_ were significantly lower than that of the SN sensor.

The results for each individual sensor module were processed via PCA, and the four noxious gases were classified and identified using PCA scatterplots. The gas sensitivity *S* was used as a major input parameter in the PCA. *S* was arranged in a specific matrix to determine whether the dual-sensor system would be applicable to the mixed gas system, with the following assumption: a sensor can have multi-selectivity to non-target gases, but no sensor has the same selectivity to all gas species. Initially, the sensing materials have non-selective adsorption sites available for both gases of a mixed gas system. For example, in a binary gas system containing oxidizing and reducing gases, both gases are adsorbed on the surface of the sensing material, and the amount of adsorbed gas molecules is proportional to the concentration of the gas in the mixed gas system. If the oxidizing gas is the major component of the binary gas system, a negligible amount of reduction of the sensing material occurs, because the reduction process that occurs via the adsorption of the reducing gas is completely canceled by the excess of the oxidation process, and the chemiresistance of the sensing material is entirely determined by the remainder of the oxidizing gas. In the case where both components are reducing or oxidizing gases, the change in the conductance of the sensing material depends on the summation of the reduction or oxidation reactions, respectively. In the PCA plot of the CO-NO_2_ system, the eight data groups (numbered 1 to 8) are clearly discriminated from each other depending on the NO_2_ concentration (Figure 6b). The gas concentration of NO_2_ appeared to significantly influence the location of the data group in the PCA plot.

**Figure 7:**
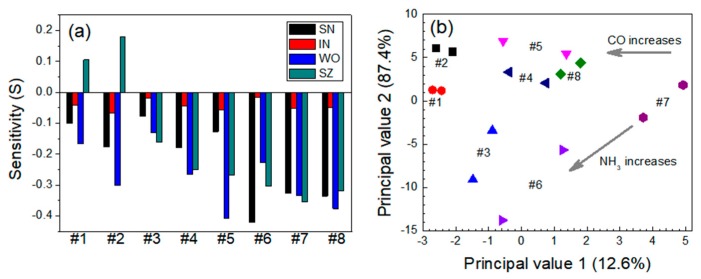
(**a**) Responses of the sensors and (**b**) PCA-based dual-gas sensor monitoring for the CO-NH_3_ system. Note that #1–#8 represent 30 ppm CO + 0 ppm NH_3_, 60 ppm CO + 0 ppm NH_3_, 0 ppm CO + 5 ppm NH_3_, 30 ppm CO + 5 ppm NH_3_, 60 ppm CO + 5 ppm NH_3_, 0 ppm CO + 10 ppm NH_3_, 30 ppm CO + 10 ppm NH_3_, and 60 ppm CO + 10 ppm NH_3_, respectively.

For the CO-NH_3_ system, as shown in Figure 7a, the responses to CO were stronger than those to NH_3_ for all sensors. Because both CO and NH_3_ are reducing gases having one or more than one lone pair of electrons, the sensitivity *S* for the mixed gas system was higher than that for each individual gas. The SN and SZ sensors were more sensitive to CO than to NH_3_ within the following test ranges: *S* = −0.179 (at CO = 30 ppm) and –0.420 (at CO = 60 ppm) for SN and *S* = −0.100 (at NH_3_ = 5 ppm) and −0.176 (at NH_3_ = 10 ppm) for SZ. The responses of the IN sensor to both gases were poor, and the sensor exhibited no selectivity. The WO and SZ sensors were sensitive to both gases, and the sensitivity to their mixture was enhanced. The sensitivity of the SN sensor was −0.420 for 60 ppm CO gas, but the sensitivity was slightly lower (*S* = −0.327) for a mixture containing 60 ppm CO and 5 ppm NH_3_. The sensitivity decreased further as the NH_3_ concentration in the mixture increased. In the corresponding PCA plot, the data points moved toward the upper-left side as the CO concentration increased, whereas they moved toward the lower-left side as the NH_3_ concentration increased (Figure 7b). The data points for the same group were diagonally dispersed, possibly owing to the higher sensitivity of the four individual sensors to the CO gas than to the other gases.

**Figure 8:**
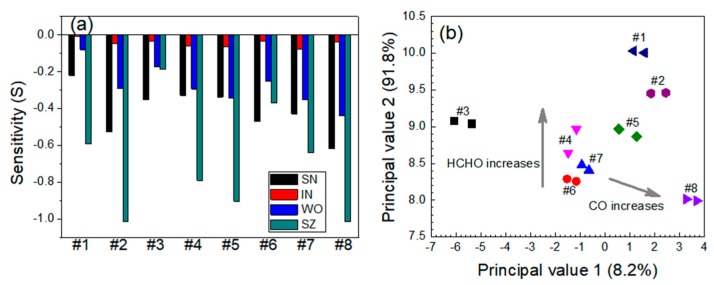
(**a**) Responses of the sensors and (**b**) PCA-based dual-gas sensor monitoring for the CO-HCHO system. Note that #1–#8 represent 30 ppm CO + 0 ppm HCHO, 60 ppm CO + 0 ppm HCHO, 0 ppm CO + 2.5 ppm HCHO, 30 ppm CO + 2.5 ppm HCHO, 60 ppm CO + 2.5 ppm HCHO, 0 ppm CO + 5 ppm HCHO, 30 ppm CO + 5 ppm HCHO, and 60 ppm CO + 5 ppm HCHO, respectively.

For the CO-HCHO system, the responses of the SN and WO sensors were almost identical for both gas species. The SZ sensor was more sensitive to HCHO than to CO. The IN sensor exhibited no responses to either gas species. The sensitivities of the four individual sensors are presented in Figure 8a. The response characteristics of the SN, WO, and SZ sensors in the CO-HCHO system exhibited almost the same pattern observed for the CO-NH_3_ system, except that the gas sensitivities were higher. The sensitivity of the SN sensor to 60 ppm CO gas was −0.470. The sensitivity was lower (*S* = −0.429) for a mixture containing 60 ppm CO and 2.5 ppm HCHO. The eight data groups shown in the PCA plots were clearly differentiated (Figure 8b). The data points moved upward as the HCHO concentration increased and toward the lower-right side as the CO concentration increased.

**Figure 9:**
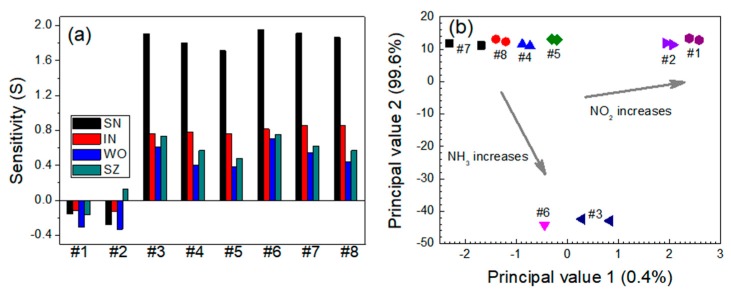
(**a**) Responses of the sensors and (**b**) PCA-based dual-gas sensor monitoring for the NO_2_-NH_3_ system. Note that #1–#8 represent 0.3 ppm NO_2_ + 0 ppm NH_3_, 0.6 ppm NO_2_ + 0 ppm NH_3_, 0 ppm NO_2_ + 5 ppm NH_3_, 0.3 ppm NO_2_ + 5 ppm NH_3_, 0.6 ppm NO_2_ + 5 ppm NH_3_, 0 ppm NO_2_ + 10 ppm NH_3_, 0.3 ppm NO_2_ + 10 ppm NH_3_, and 0.6 ppm NO_2_ + 10 ppm NH_3_, respectively.

For the NO_2_-NH_3_ system, the responses to NO_2_ gas were stronger than those to NH_3_ (Figure 9a). The sensitivities of the SN sensor increased with an increasing NO_2_ concentration at a constant NH_3_ concentration and decreased with an increasing NH_3_ concentration in the mixture. The sensitivities to both gases were positive (*S* > 0) within the test ranges. The IN sensor exhibited a selective response to NO_2_ and was hardly sensitive to NH_3_ gas. As the NH_3_ gas concentration increased, the IN sensor exhibited a slight decrease in sensitivity to the NO_2_ gas in the NO_2_-NH_3_ mixed gas system. The WO sensor exhibited similar behavior to the IN sensor; i.e., it was far more sensitive to the NO_2_ gas. Figure 9b shows the PCA plots for the NO_2_-NH_3_ gas mixtures. The data points were segregated into eight distinct, non-overlapping groups. As the NO_2_ concentration increased, the data points moved toward the upper-right side. The influence of NH_3_ was smaller than that of NO_2_, but the NH_3_ gas reduced principal value 2.

**Figure 10:**
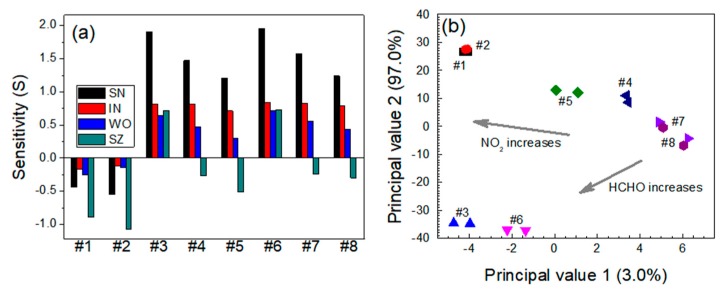
(**a**) Responses of the sensors and (**b**) PCA-based dual-gas sensor monitoring for the NO_2_-HCHO system. Note that #1–#8 represent 0.3 ppm NO_2_ + 0 ppm HCHO, 0.6 ppm NO_2_ + 0 ppm HCHO, 0 ppm NO_2_ + 2.5 ppm HCHO, 0.3 ppm NO_2_ + 2.5 ppm HCHO, 0.6 ppm NO_2_ + 2.5 ppm HCHO, 0 ppm NO_2_ + 5 ppm HCHO, 0.3 ppm NO_2_ + 5 ppm HCHO, and 0.6 ppm NO_2_ + 5 ppm HCHO, respectively.

The sensor responses to NO_2_ gas were usually stronger than those to HCHO gas in the NO_2_-HCHO system, except that the SZ sensor was more susceptible to HCHO gas (Figure 10a). The sensitivity of the SN and WO sensors increased with the increasing NO_2_ concentration and decreased with the increasing HCHO concentration. The IN sensor exhibited a selective response to NO_2_ but no sensitivity to HCHO. More precisely, the IN sensor exhibited a slight decrease in sensitivity to NO_2_ as the HCHO concentration increased in the gas mixture. The WO sensor exhibited similar behavior to the IN sensor but better sensitivity to NO_2_. The SZ sensor responses to HCHO were stronger than those to NO_2_ in the gas mixture. Figure 10b shows the PCA plots for the NO_2_-HCHO system. Principal value 1 was small in the presence of NO_2_ gas. Overlapping groups appeared in two areas. One was observed in cases where the NO_2_ concentrations were 0.3 and 0.6 ppm, in the absence of HCHO. Another was observed for the gas mixtures, i.e., 0.3 ppm NO_2_ + 5.0 ppm HCHO and 0.6 ppm NO_2_ + 5.0 ppm HCHO. These results might be due to different sensitivities between SZ and the other sensors.

**Figure 11:**
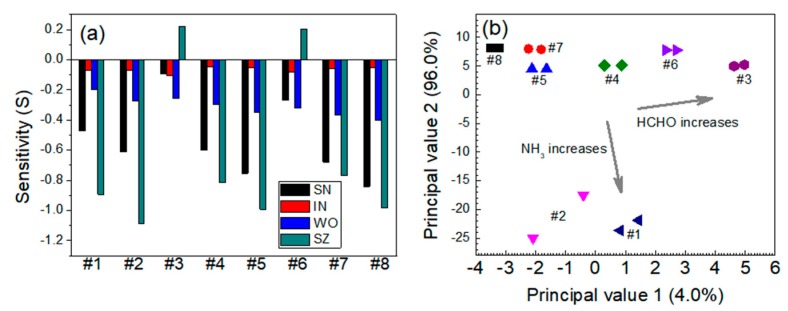
(**a**) Responses of the sensors and (**b**) PCA-based dual-gas sensor monitoring for the NH_3_-HCHO system. Note that #1–#8 represent 5 ppm NH_3_ + 0 ppm HCHO, 10 ppm NH_3_ + 0 ppm HCHO, 0 ppm NH_3_ + 2.5 ppm HCHO, 5 ppm NH_3_ + 2.5 ppm HCHO, 10 ppm NH_3_ + 2.5 ppm HCHO, 0 ppm NH_3_ + 5 ppm HCHO, 5 ppm NH_3_ + 5 ppm HCHO, and 10 ppm NH_3_ + 5 ppm HCHO, respectively.

For the NH_3_-HCHO system, the sensor responses to HCHO were significantly stronger than those to NH_3_ gas (Figure 11a). The behavior of the SN, WO, and SZ sensors was almost identical to that for the other reducing agent systems (CO-NH_3_ and CO-HCHO); i.e., all the sensors except the IN sensor were highly sensitive to each gas and gas mixture. The SZ sensor responses to HCHO were stronger than those to NH_3_, and no synergistic effect of the SN sensor was observed. In the PCA plot for the NH_3_-HCHO system, the data points moved slightly toward the upper-right side as the HCHO concentration increased and moved toward the lower side as the NH_3_ concentration increased (Figure 11b). This result might be caused by an imbalanced sensitivity or susceptibility to the gases in the mixture. The higher sensitivity to HCHO indicates that NH_3_ mainly affected the positions of the data points in the PCA plots for the NH_3_-HCHO system.

## 4. Discussion

Metallic nanopowder-added gas-sensing nanocomposites such as Pd-doped SnO_2_, In_2_O_3_, Ru-doped WO_3_, and SnO_2_-doped ZnO were synthesized using a sol-gel method and used to develop a MEMS-based dual-sensor system for monitoring two major exhaust gases (CO and NO_2_) and two odorous gases (NH_3_ and HCHO). These gases appreciably affect the indoor air quality, i.e., CO is commonly produced during gas heating, cooking for flame foods, lighting with an oil lamp, etc. NH_3_ emissions in a house mostly come from the bathroom. Carcinogenic HCHO can be more concentrated in indoor air than the outdoor because a variety of composite wood products are used indoors such as medium-density fiberwoods, plywood boards, and particle boards. The operating temperatures for the four single sensor modules were optimized to obtain the best sensitivity and selectivity to the target gases. While the IN sensor could selectively detect NO_2_, the SN sensor was sensitive to all target gas species. Therefore, NO_2_ can be selectively identified by coupling the SN and IN sensors, even though their output signals are insufficient to identify the other gases. Because the WO and SZ sensors can detect all target gases with excellent sensitivity (but poor selectivity), two dual-sensor arrays would be sufficient to clearly identify any mixed gases among the four target gases. PCA-based characterization of the dual-sensor systems for the detection of multi-component toxic gases indicated that the dual-sensor system follows conventional adsorption and desorption mechanism of the n-type MOS sensing material. We believe that the MEMS-based dual-sensor array is highly effective for monitoring indoor air quality in real-time.

## Figures and Tables

**Table 1:** Chemiresistive responses of each individual sensor to the two-component mixed gas systems.

Sensor	Gas Mixtures					
	CO + NO_2_	CO + NH_3_	CO + HCHO	NO_2_ + NH_3_	NO_2_ + HCHO	NH_3_ + HCHO
SN	NO_2_	CO	both	NO_2_	NO_2_	HCHO
IN	NO_2_	None	none	NO_2_	NO_2_	none
WO	NO_2_	CO	both	NO_2_	NO_2_	HCHO
SZ	NO_2_	CO	HCHO	NO_2_	HCHO	HCHO
